# Disorganized attachment in infancy: a review of the phenomenon and its implications for clinicians and policy-makers

**DOI:** 10.1080/14616734.2017.1354040

**Published:** 2017-11-02

**Authors:** Pehr Granqvist, L. Alan Sroufe, Mary Dozier, Erik Hesse, Miriam Steele, Marinus van Ijzendoorn, Judith Solomon, Carlo Schuengel, Pasco Fearon, Marian Bakermans-Kranenburg, Howard Steele, Jude Cassidy, Elizabeth Carlson, Sheri Madigan, Deborah Jacobvitz, Sarah Foster, Kazuko Behrens, Anne Rifkin-Graboi, Naomi Gribneau, Gottfried Spangler, Mary J Ward, Mary True, Susan Spieker, Sophie Reijman, Samantha Reisz, Anne Tharner, Frances Nkara, Ruth Goldwyn, June Sroufe, David Pederson, Deanne Pederson, Robert Weigand, Daniel Siegel, Nino Dazzi, Kristin Bernard, Peter Fonagy, Everett Waters, Sheree Toth, Dante Cicchetti, Charles H Zeanah, Karlen Lyons-Ruth, Mary Main, Robbie Duschinsky

**Affiliations:** ^a^ Department of Psychology, Stockholm University, Stockholm, Sweden; ^b^ Institute of Child Development, University of Minnesota, Minneapolis, MN, USA; ^c^ Department of Psychological and Brain Sciences, University of Delaware, Newark, NY, USA; ^d^ Department of Psychology, University of California, Berkeley, CA, USA; ^e^ Psychology Department, The New School, New York, NY, USA; ^f^ Department of Psychology, Education, and Child Studies, Erasmus University Rotterdam, Rotterdam, The Netherlands; ^g^ Department of Public Health & Primary Care, University of Cambridge, Cambridge, UK; ^h^ Clinical Child and Family Studies and Amsterdam Public Health, Vrije Universiteit Amsterdam, Amsterdam, The Netherlands; ^i^ Research Department of Clinical, Educational and Health Psychology, University College of London, London, UK; ^j^ Child and Family Studies, Leiden University, Leiden, The Netherlands; ^k^ Psychology Department, University of Maryland, Washington DC, USA; ^l^ Department of Psychology, University of Calgary, Calgary, Canada; ^m^ Department of Psychology, The University of Texas, Austin, TX, USA; ^n^ Faculty of Health and Life Sciences, University of Northumbria, Newcastle, UK; ^o^ Department of Social and Behavioral Sciences, SUNY Polytechnic Institute, Utica, NY, USA; ^p^ the Neurodevelopmental Research Center, the Singapore Institute for Clinical Sciences, Singapore, Singapore; ^q^ Developmental and Educational Psychology, University of Erlangen-Nuremberg, Erlangen, Germany; ^r^ Department of Pediatrics, Weill Cornell Medical College, New York, NY, USA; ^s^ Psychology Department, St Mary’s College of California, Moraga, NY, USA; ^t^ Center on Human Development and Disability, University of Washington, Seattle, MA, USA; ^u^ the Department of Human Development and Family Sciences, University of Texas at Austin, Austin, TX, USA; ^v^ Child and Adolsecent Mental Health Service, Trafford Children and Young Peoples Service, Manchester, UK; ^w^ Minnesota Institute for Contemporary Psychotherapy and Psychoanalysis, Minneapolis, MI, USA; ^x^ Department of Psychology, University of Western Ontario, London, ON, Canada; ^y^ University of Western Ontario, London, ON, Canada; ^z^ T. Denny Sanford School of Social & Family Dynamics, Arizona State University, Tempe, AZ, USA; ^aa^ School of Medicine, University of California, Los Angeles, CA, USA; ^ab^ Department of Dynamic and Clinical Psychology, Sapienza University of Rome, Rome, Italy; ^ac^ Department of Psychology, Stony Brook University, Stony Brook, NY, USA; ^ad^ The Mt. Hope Family Center, University of Rochester, Rochester, NY, USA; ^ae^ Institute of Infant and Early Childhood Mental Health, Tulane University, New Orleans, LA, USA; ^af^ Department of Psychiatry, Harvard Medical School, Boston, MA, USA

**Keywords:** Disorganized attachment, infancy, attachment-based interventions, maltreatment, attachment disorder

## Abstract

Disorganized/Disoriented (D) attachment has seen widespread interest from policy makers, practitioners, and clinicians in recent years. However, some of this interest seems to have been based on some false assumptions that (1) attachment measures can be used as definitive assessments of the individual in forensic/child protection settings and that disorganized attachment (2) reliably indicates child maltreatment, (3) is a strong predictor of pathology, and (4) represents a fixed or static “trait” of the child, impervious to development or help. This paper summarizes the evidence showing that these four assumptions are false and misleading. The paper reviews what is known about disorganized infant attachment and clarifies the implications of the classification for clinical and welfare practice with children. In particular, the difference between disorganized attachment and attachment disorder is examined, and a strong case is made for the value of attachment theory for supportive work with families and for the development and evaluation of evidence-based caregiving interventions.

This review paper represents a broadly held consensus concerning what we currently understand about disorganized infant attachment and its implications across clinical and child welfare practices. Our hope is that this review will prove to be useful both in supporting best practice and in highlighting the gaps that occasionally surround the concept of attachment disorganization, particularly between basic theory and research on the one hand and their applications in clinical and child welfare practice on the other.

## Summary of 10 topics to be elaborated upon in this review

1. The disorganized infant attachment category can be assigned by trained and certified coders to infant behavior (age 12–20 months) in the Strange Situation when there is a sufficient fit to one or several of the behaviors listed under Main and Solomon’s (1986, 1990) seven thematic headings. Persons interested in seeking training to code disorganized attachment should go directly to attachment-training.com.
2. *Behaviors* from Main and Solomon’s list can occur for a variety of reasons. They are quite common at low levels in the Strange Situation among infants from populations facing adversity. Only when these behaviors are sufficiently intense can a classification of disorganized *attachment* be assigned.
3. Disorganized infant attachment is more common among maltreated infants but does not necessarily indicate maltreatment. As it stands, the disorganized attachment classification cannot be used to screen for maltreatment. This is because a significant proportion of maltreated infants do not show disorganized attachment in the Strange Situation, and many infants showing disorganized attachment in the Strange Situation have not been maltreated. Thus, there are other pathways to disorganized attachment besides maltreatment.
4. These other pathways to disorganized attachment may feature a parent’s unresolved trauma or loss. Such experiences may lead a parent to display subtly frightening, frightened, or dissociative behaviors toward their infant. Other contributing factors for at least some of the behaviors used to classify disorganized attachment may include the infant’s genetic and temperamental susceptibility. In addition, major or repeated separations can cause disorganized behaviors. Therefore, for children in placement who undergo such separations, disorganized behaviors may be especially misleading regarding the usual state of child–parent attachment.
5. Research at the group level has established disorganized infant attachment as a small-moderate predictor for the development of social and behavior problems. However, disorganized infant attachment does not inevitably cause later problems. When infants classified as disorganized do develop such problems, this may be the result of a continuation of difficult life circumstances rather than solely an effect of early disorganized attachment.
6. Disorganized infant attachment is *not* a validated individual-level clinical diagnosis. This is unlike the two attachment-related disorders included in the DSM/ICD diagnostic systems, developed for the clinical categorization of young children reared in conditions of severe neglect. These disorders are expressed across contexts – that is, they are present in multiple settings and with different adults. In contrast with those clinical disorders, disorganized infant attachment is not a fixed property of the individual child but is relationship specific.
7. It is crucial to recognize that some misapplications of ideas relating to disorganized attachment have accrued in recent years (e.g. in the context of child removal decisions). Such misapplications can result from erroneous assumptions that (1) attachment measures can be used as definitive assessments of the individual in forensic/child protection settings and that disorganized attachment (2) reliably indicates child maltreatment, (3) is a strong predictor of pathology, and (4) represents a fixed or static “trait” of the child (i.e. is not altered by development or changes in available family support). These are myths or exaggerations regarding disorganized attachment, without support from research evidence.
8. Misapplications are likely to selectively harm already underprivileged families (e.g. those raised by parents in socioeconomic adversity or with functional impairments). Misapplications may violate children’s and parents’ human rights and represent discriminatory practice against minorities in need of social and material support. Child removal from his/her original family can never be justified solely by the child’s display of disorganized attachment to a caregiver.
9. It is important to recognize that there is robust evidence that both (1) attachment-based interventions and (2) naturalistically occurring reparative experiences (stable, safe, and nurturing relationships) can break intergenerational cycles of abuse and lower the proportion of children with disorganized attachment.
10. The real practical utility of attachment theory and research resides in supporting understanding of families and in providing evidence-based interventions. In this way, attachment theory, assessments, and research can have major roles to play in *clinical formulation* and *supportive* welfare and clinical work. We offer key examples of interventions in the section “Attachment-based clinical interventions”.

## Introduction

It is not uncommon to hear clinicians, practitioners, and policy makers speak about disorganized child–parent attachment. The concept of disorganized infant attachment was initially proposed to account for conflicted, disoriented, or fearful behavior shown by infants toward their caregiver in a laboratory setting (Main & Solomon, 1986, 1990). This work has led to an array of empirical research that has tested key assumptions about the causes and implications of disorganized attachment (Madigan et al., 2006; van IJzendoorn, 1995) and now provides the evidence base for intervention programs (see Facompré, Bernard, & Waters, 2017; Steele & Steele, in press). There has also been appeal to the disorganized attachment classification in the context of custodial placements and child welfare assessments.

Sometimes thinking about disorganized attachment has supported excellent practice, by informing clinical and child welfare practitioners in reflecting on family needs and service provision in the context of a variety of other forms of assessment. However, regrettably, it has also been evident that use of the disorganized attachment classification has sometimes reflected serious misapplications of attachment theory and related research (see discussions by Alexius & Hollander, 2014; Granqvist, 2016). We sympathize wholeheartedly with practitioners, who often find themselves confronted by challenging and important tasks but without the resources required to carry out those tasks with sufficient time and rigor, and without required training in attachment. We recognize that this *structural* shortcoming, which places practitioners in need of a shortcut, is likely at the core of the problems to be discussed (Boris & Renk, 2017).

This consensus statement has three aims. Misinformation about the classification is truly widespread. So, our first aim is to *characterize and explain the concept of disorganized infant attachment*. Second, in the service of preventing future misuse, we *identify misconceptions and misapplications of the idea of disorganized attachment*, especially in the context of assessment. Third, we *provide recommendations for the relevance of attachment theory and the value of evidence-based applications of attachment theory* for practitioners reflecting on the best way to help families; this is where the real practical utility of attachment theory resides. We start by describing secure and organized-insecure forms of infant attachment in order to situate disorganized attachment and its relevance for practitioners.

### What are secure and organized-insecure attachment?

Infant patterns of attachment were identified in a formal laboratory situation known as the Strange Situation, developed by Mary Ainsworth (Ainsworth, Blehar, Waters, & Wall, 1978). Ainsworth and subsequent researchers looked at how the infant explored an unfamiliar room and its toys, and how the infant responded to the caregiver when alarmed or distressed by two brief separations. What Ainsworth and colleagues termed “secure attachment” has two aspects. First, it refers to a basic confidence that the infant has in the caregiver to be responsive and comforting when the infant is alarmed or stressed. Second, secure attachment also means that children have confidence in their caregiver as a secure base from which to explore, meaning that during their ventures and play, they expect support, not interference, and can attend fully to exploration when feeling calm. Ainsworth et al.’s (1978) home observations revealed that the confidence of securely attached infants to strike a balance between attachment and exploration was based on experiencing responsive care at home. Later, research and intervention work with parents have supported this conclusion (e.g. Powell, Cooper, Hoffman, & Marvin, 2013).

Yet, not all infants show this degree of confidence. For example, some infants experience consistent rebuff of distress signals and approach behavior by their caregiver. Typically, such infants develop an “insecure-avoidant” attachment pattern (Ainsworth et al., 1978), in which their response to alarm, where possible, is to shift their attention toward exploration of the environment at the expense of communication of their feelings to their caregiver. In doing so, they are thought to be responding to the caregiver’s discomfort with close contact. In other words, these infants minimize their attention to attachment-related information that might otherwise lead them to approach the caregiver, as this retains the availability of the caregiver (Main, 1990). As long as the caregiver continues to provide reasonable protection and monitoring in the context of more emotional distance, this adjustment allows the infant to achieve an organized, workable attachment strategy.

Other infants may have experienced unreliable caregiver responsiveness when they make their desire for comfort known, leading them to be highly vigilant about their attachment figure’s accessibility. Typically, such infants develop “insecure-ambivalent/resistant” attachment, seen as inconsolable distress and/or anger in the Strange Situation, which retains the attention of the caregiver (Ainsworth et al., 1978). Even in situations without significant alarming cues, these infants may engage in attachment behavior such as clinging to the caregiver at the expense of play, or mixing whiny or angry behavior with distress. In other words, they maximize their attention to attachment-related information (Main, 1990) to such an extent that it interferes with exploration. Again, to the extent that the caregiver does respond by giving attention, the child’s heightening of attachment behaviors can result in an organized workable attachment strategy. These three basic patterns (i.e. secure, insecure-avoidant, insecure-ambivalent/resistant) serve as the background for our understanding of disorganized attachment. Importantly, these infant–caregiver patterns have been shown to be *relationship specific*: this means that an infant/toddler may well show one pattern to a particular caregiver, and a different pattern to another caregiver – as practitioners may well have observed in informal situations.

### What is disorganized attachment?

The idea of disorganized attachment (Main & Solomon, 1986, 1990) arose out of a growing awareness amongst researchers that not all infant responses in the Strange Situation could be placed in the original patterns defined by Ainsworth (see Duschinsky, 2015). On reunion with their caregiver in the Strange Situation, some infants were seen to display various conflicted, disoriented, or fearful behaviors. The term “disorganized” itself can be a little confusing, since in ordinary language, the word can mean “random.” However, in fact, Main and Solomon (1986, 1990) identified specified classes of behaviors that – if seen at sufficient intensity and in the presence of the parent in the Strange Situation – could lead to a disorganized attachment classification. The classes were (1) sequential and (2) simultaneous display of contradictory behavior patterns; (3) undirected, misdirected, incomplete, and interrupted movements and expressions; (4) stereotypies, asymmetrical, and mistimed movements and anomalous postures; (5) freezing, stilling, and slowed movements and expressions; (6) direct indices of apprehension regarding the parent; and (7) Direct indices of disorganization and disorientation. It is the intensity of the display of conflict, disorientation or fear, and the extent to which this disrupts a child’s attachment strategy that lead to a disorganized attachment classification. For this reason, infants classified as disorganized are also given an alternate “best-fitting” (secondary) organized category as well (e.g. disorganized/avoidant attachment). This convention has led to a particularly marked decrease in primary resistant category assignments; when resistance is present to a significant degree, the child also often receives a disorganized classification due to marked display of disorganized behaviors (van IJzendoorn, Schuengel, & Bakermans-Kranenburg, 1999).

Experienced coders of the Strange Situation know that it is fairly common for infants to show a certain amount of the behaviors listed by Main and Solomon, and even more so for infants drawn from populations facing adversity. As a result, seeing one or another example of disorganized infant behavior in the Strange Situation is not, in itself, sufficient for a disorganized classification unless certain thresholds of intensity are met (Main & Solomon, 1990). Recognizing such thresholds forms a core part of the accredited training and reliability process (see http://attachment-training.com/at/home/training/). Moreover, an infant may display disorganized attachment with one parent and yet organized, even secure, attachment with the other (e.g. Steele, Steele, & Fonagy, 1996). Thus, disorganized attachment is not a fixed property or trait of the individual child but tends to be relationship specific. Even within a child’s relationship with a particular caregiver, disorganized attachment displays only *modest* stability over time (van IJzendoorn et al., 1999).

Finally, Main and Solomon (1990) advise that the disorganized attachment coding system should not be used for infants above 20 months, since after that children generally develop more sophisticated strategies for coping with caregiver behavior, and may therefore no longer show the indexed disorganized behaviors. The focus of this consensus statement is on infancy. However, it can be briefly noted that with growing cognitive and social abilities, formerly disorganized children may adopt controlling (caregiving or punitive) strategies to help manage dysregulated, unpredictable, or frightening caregiving environments (e.g. Main & Cassidy, 1988; Solomon, George, & De Jong, 1995; compare with Crittenden, 2016). When assessed via representational (e.g. semi-projective interviews) rather than behavioral methods, these children’s attachment representations are nonetheless likely to express fearful elements (e.g. catastrophic fantasies; Main, Kaplan, & Cassidy, 1985). It should be noted, however, that the infant disorganized attachment classification under discussion here, and its connection with fearful attachment representations, should not be confused with constructs from the self-report romantic attachment literature (e.g. “fearful adult attachment style”). There is as yet little evidence that they refer to the same thing, even if the self-report assessments appeal to some of the same concepts such as “attachment” and “fear” (see Bartholomew & Horowitz, 1991; Bifulco, Jacobs, Bunn, Thomas, & Irving, 2008; Rholes, Paetzold, & Kohn, 2016).

### What are the psychological processes behind disorganized attachment?

Both outside and inside the Strange Situation, it is important to note that the *behaviors* listed by Main and Solomon can occur for a variety of reasons unrelated to the history of the relationship with the caregiver. For example, exhaustion, illness, irresolvable pain, neurological disturbance, and excessive situational stress (e.g. brief parental unavailability in a busy and noisy supermarket) might all lead an infant to show some of the behaviors that Main and Solomon list, for instance tension behaviors like stereotypies. However, that would not be the kind of disorganization that we are concerned with when we are thinking about disorganized *attachment*.

Disorganized attachment is coded in a standardized procedure in which a child has been moderately alarmed, and where the display of the behaviors that Main and Solomon list can be assumed to reflect, to varying degrees, a disruption of the child’s attachment response to their caregiver in the context of that alarm. There is consensus in the field that this can occur for a variety of reasons. For example, some infants may, because of dispositional or neurological factors, have more difficulty than others in achieving a single strategy for utilizing the caregiver as a safe haven. This could increase their odds of showing conflicted behavior in the Strange Situation, though not necessarily overtly frightened responses to the caregiver (e.g. Padrón, Carlson, & Sroufe, 2014; Spangler, Femmer-Bombik, & Grossmann, 1996). Relatedly, the behaviors listed by Main and Solomon (1986, 1990) may well be seen in infants who would *not* receive a disorganized classification by experienced coders. For instance, stereotypic behaviors would be discounted by trained coders if they suspect that the infant has a neurological or developmental disorder (e.g. Capps, Sigman, & Mundy, 1994; Rozga et al., in press; Dozier & Bernard, 2017).

However, as mentioned in the previous section, a classification of disorganized attachment with one caregiver does not have an association with disorganized attachment with another (van IJzendoorn et al., 1999). This suggests that much of the variance can be accounted for by relationship-specific factors, or by interactions between infant disposition and the caregiving environment. For example, one study by Bakermans-Kranenburg and van IJzendoorn (2007) found that a genetic marker (DRD4 7-repeat polymorphism) increased the risk of developing disorganized attachment when *combined* with environmental risk. Similarly, Spangler, Johann, Ronai, and Zimmermann (2009) found an association between a serotonin transporter gene (the short allele variation of the 5-HTTLPR) and attachment disorganization when maternal responsiveness was low, but not when responsiveness was high.

In considering the kinds of caregiving behavior that tend to be associated with infant disorganized attachment, it has been theorized that infants may show disorganized attachment in the Strange Situation because they have had experiences of their caregiver as a regular source of alarm. Alarming behavior can take several forms, including subtly frightening or frightened parental behaviors (e.g. Hesse & Main, 2006), states of mind that leave the caregiver psychologically unavailable to the child, threats of harm, or even unusually extended absences (Solomon & George, 2011). A child may also be expected to associate alarm with a caregiver who they have seen subjected to partner violence (Lieberman & Amaya-Jackson, 2005). Experiences of the caregiver as a source of alarm can lead to a disposition to move away, withdraw, or flee from the caregiver when future experiences of alarm occur. However, the attachment response directs an infant to seek safety from their caregiver. The result is a paradoxical situation for the infant (Duschinsky, Main, & Hesse, in press; Hesse & Main, 2000). Albeit to varying degrees, the different behaviors listed by Main and Solomon (1990) can be regarded as consequences of a tendency to *approach* the attachment figure and a simultaneous tendency to *move away* from the attachment figure. This is why most forms of disorganized attachment appear as conflicted, confused, and/or apprehensive behavior toward the caregiver, since these qualities can characterize a child’s paradoxical situation (Hesse & Main, 2006; Solomon, Duschinsky, Bakkum, & Schuengel, in press).

It might seem strange that a child who is alarmed by the caregiver is nonetheless motivated to approach the caregiver, for instance after a brief laboratory separation. However, the child’s attachment system is one of multiple behavioral systems (including the caregiving system, the fear system) which, when activated, motivate the organism to achieve a certain goal. The attachment system is biologically channeled to make the child want to approach a familiar caregiver when there are cues to danger in the environment or when he or she has been unexpectedly separated from that caregiver. This concrete prediction was one of the core elements of John Bowlby’s (1969) theory and part of what has made attachment theory so compelling and powerful as a research tool and basis for thinking about clinical interventions. Hesse and Main (2000) reasoned that, at an evolutionary level, proximity to even an alarming caregiver would likely have helped a human infant survive, given that infants are unable to fend for or regulate themselves.

This conclusion is supported by findings that a number of (often subtle) frightening, frightened, and dissociative caregiver behaviors are associated with elevated rates of infant D attachment (e.g. Abrams, Rifkin, & Hesse, 2006; Madigan et al., 2006; Schuengel, Bakermans-Kranenburg, & van IJzendoorn, 1999). These are especially common in parents who are still troubled by their own experiences of loss or trauma to the point that this intrudes into their thinking and behavior (what is known as “unresolved loss or trauma”). For instance, Hughes, Turton, McGauley, and Fonagy (2006) found that a majority of infants to mothers who had unresolved loss regarding a previous still-birth received a classification of disorganized attachment. There is no expectation that these mothers are abusive to their infants, but women who have had this terribly sad thing happen may remain very troubled by the experience in a way that impacts their caregiving of a next-born child.

Even though the caregiver may not be doing anything abusive to the infant, a caregiver who is him- or herself in some way alarming to the child can create a paradoxical predicament for a child because the parent who is the source of safety is then also the source of alarm, increasing the chances of disorganized attachment being displayed by the infant in the Strange Situation. A parent who is experiencing an acute combination of socioeconomic risks or a parent unresolved with regard to loss or trauma (like sexual or physical abuse in their own history) may be a sensitive, non-abusive caregiver. However, they may nonetheless still harbor frightening ideas, experience dysregulating emotions, and be prone to enter segregated (mildly dissociative) mental states. When the parent shows fear or threat in these states, he or she is theorized to be alarming to the infant (e.g. looming into the baby’s face; Jacobvitz, Leon, & Hazen, 2006). The expression of such behaviors by the caregiver can, in many cases, be outside the conscious awareness of the individual. It is important to recognize that “blaming” these caregivers for their behavior, or engaging in punitive responses to them, is therefore mistaken and likely counterproductive. As we will elaborate further in the sections that follow, understanding the development of attachments changes the clinical imperative from retribution for errors to efforts in assisting parents to adopt caregiving behaviors that promote feelings of safety and security in the child.

### Can disorganized attachment be used to infer children’s experiences with caregivers and forecast their developmental prospects?

Practitioners will wish to consider what they *can* infer from a classification of disorganized attachment. Even if told by a certified attachment coder that an infant’s behavior in the Strange Situation has received a disorganized classification, a practitioner can only infer that this infant has experienced alarm in relation to the caregiver for some reason and has a somewhat higher risk of social–emotional developmental difficulties. These two inferences suggest priorities and areas for support for the caregiver, but with only broad brushstrokes in the absence of additional assessment. Accordingly, disorganized attachment with a particular caregiver is best thought of as a risk factor for later social and externalizing problems, contributing as one factor among many others (Fearon, Bakermans‐Kranenburg, van IJzendoorn, Lapsley, & Roisman, 2010; Groh et al., 2014; Groh, Fearon, IJzendoorn, Bakermans‐Kranenburg, & Roisman, 2017). The average effect size linking infant disorganized attachment with a particular caregiver to later behavior problems is small to moderate (for discussion of what effect sizes mean in practice, see Ferguson, 2009). In other words, a child assigned a disorganized classification is not necessarily expected to develop behavior problems. Additionally, when infants classified as disorganized do develop such problems, this may also be the result of a continuation of difficult life circumstances rather than solely an effect of early disorganized attachment (Sroufe, 2016).

Similarly, assessments of disorganized attachment are reasonably good at discerning infants’ experiences of caregiving at the *group level*, but they have not been validated for making inferences about an *individual* infant’s experience. Disorganized classifications are typically based on an infant’s behavior at a single point in time during a brief assessment; it cannot be taken to be a true reflection of the infant’s attachment relationship in every case. In research, direct effects of early relationships on later outcomes are probabilistic and are more in evidence when cumulative assessments are used rather than a single measure at a particular time (Sroufe, Egeland, Carlson, & Collins, 2005). Researchers and clinicians can increase the validity of conclusions about an infant’s experiences by carefully compiling a body of observations and information about the history of that particular parent–infant dyad. This should include, above all, what goes on in the home, as well as consideration of the wider context supporting or depleting the emotional and material resources available both to the caregiver and to the infant. Naturally, for a clinician who encounters an infant exhibiting disorganized behaviors toward a caregiver, it is perfectly reasonable to use this as a way of trying to understand how to intervene to enhance the relationship. If frightening or frightened behaviors are evident, for example, working with a parent to eliminate these is indicated (see also the “Attachment in clinical assessment and formulation” section). However, this is quite different from using observations of disorganized behaviors prognostically.

Maltreatment was identified quite early as one possible cause of disorganized attachment in relation to the caregiver (Carlson, Cicchetti, Barnett, & Braunwald, 1989). However, there are two important qualifications that need to be made in relation to this statement. A first is that a significant proportion of maltreated infants do not receive a classification of disorganized attachment with the maltreating caregiver in the Strange Situation. Although meta-analytic data show that maltreated infants are much more likely to receive a disorganized attachment classification than infants drawn from samples with few risk factors (Cyr, Euser, Bakermans-Kranenburg, & van IJzendoorn, 2010), such data also show that a large proportion of maltreated infants do not receive a disorganized classification (van IJzendoorn et al., 1999).

The second qualification is that there are other pathways to disorganized attachment besides maltreatment. Of particular relevance here for the issue of specificity is that for infants from families experiencing five or more socioeconomic risk factors, rates of disorganized attachment are also high, and similar in prevalence to samples of infants known to be maltreated (Cyr et al., 2010). Cyr and colleagues argue that such findings do not necessarily imply that these socioeconomic risk families with infants classified as disorganized all engage in maltreatment. Rather, the authors argue that the accumulation of socioeconomic risks leads to a disorganized attachment classification by creating a frightening and distressing situation for a caregiver who might otherwise be able to provide adequate care (Cyr et al., 2010).

### Can the disorganized attachment classification be used to screen for maltreatment?

We know that disorganized attachment *is* overrepresented in maltreatment samples compared to samples from the general population. Therefore, it may be tempting to ask whether assessments of disorganized attachment might be used at least as a “proxy” or screening tool for maltreatment? Recommendations have been offered to practitioners (e.g. Building Great Britons, 2015; Wilkins, 2012), suggesting that the disorganized attachment classification offers child protection workers a way to cut through the particularities of potential maltreatment cases, seeing through to the needs of a child and their likely future outcomes. In a resource-strapped context, such a prospect is understandably appealing; and child protection workers report that it has helped them in making assessments of families, made decision-making easier, and helped them distinguish between abused and non-abused children (Wilkins, 2017). However, caution is warranted here.

In order to understand the value and limitations of disorganized attachment, it may be helpful to identify some relevant key requirements of screening instruments. Screening instruments require adequate sensitivity (i.e. high probability of detecting a phenomenon) and specificity (i.e. accuracy in detecting nonclinical phenomena) to be useful. The disorganized attachment classification has insufficient sensitivity and specificity for screening for maltreatment (Granqvist et al., 2016; Main, Hesse, & Hesse, 2011). In addition to this, there is the need for proper training to code disorganized attachment (http://attachment-training.com/at/), and the child protection workers interviewed by Wilkins (2017) had not received accredited reliability to do so. We know, of course, that child protection workers can and do make fine coders of disorganized attachment. However, even when accredited reliability is in place, the results of any assessment of attachment should be used to inform clinical formulation (to be discussed further shortly), rather than as a definitive means of assessment for maltreatment or developmental risk.

### Disorganized attachment vis-à-vis attachment disorder

How does disorganized attachment relate, then, to “attachment disorder,” which is an individual-level clinical diagnosis? To be clear, they are completely different things. Disorganized attachment is a technical, research-based term that comes from coding infant behaviors in a specific laboratory situation, the Strange Situation, at age 12–20 months. No replicated research has yet established that children assigned a disorganized classification in the Strange Situation show the behaviors listed by Main and Solomon in naturalistic settings, such as the child’s home. Conversely, children who display disorganized behaviors in naturalistic settings may or may not receive a classification of disorganized attachment in the Strange Situation (Schuengel, van IJzendoorn, Bakermans-Kranenburg, & Blom, 1998).

In contrast, the attachment-related disorders listed in psychiatric diagnostic systems such as the Diagnostic and Statistical Manual (DSM; American Psychiatric Association, 2013) refer to clusters of behaviors first described among children reared from infancy in orphanages, without biological parents present. In the DSM, there are two attachment-related diagnoses, and both are strongly associated with experiences of extreme social neglect, capturing “distinctive patterns of aberrant attachment and social behaviors in young children who are socially neglected or are being raised in environments that limit opportunities to form selective attachments” (Zeanah et al., 2016, p. 990). The first is reactive attachment disorder (RAD), which is assigned to children who are very inhibited or withdrawn from their caregivers and who do not show proximity seeking or contact maintenance to the caregivers, even when the children display high distress. The second attachment-related diagnosis in the DSM is disinhibited social engagement disorder (DSED; formerly RAD subtype II: disinhibited). It is characterized by failure to show a preference for familiar caregivers, even when the child is frightened or distressed. In an important study, Woolgar and Baldock (2015) reported data suggesting the widespread overuse of the attachment disorder diagnoses for children who do not meet the DSM criteria. Both attachment disorders should only be assigned to children who meet the diagnostic criteria before the age of 5 years and after 9 months of age (i.e. when an attachment has usually formed).

Unlike disorganized attachment, which is a response to a particular caregiver in a specific situation, both attachment-related disorders signify behaviors that are understood to permeate many naturalistic situations in the child’s life. While an association between disorganized attachment with the primary caregiver in infancy and DSED has been reported (Gleason et al., 2014; Lyons-Ruth, Bureau, Riley, & Atlas-Corbett, 2009; Vorria et al., 2003), disorganized attachment is much more prevalent than either of the two attachment-related disorders and cannot be equated with them. For instance, Smyke, Zeanah, Fox, Nelson, and Guthrie (2010) found that rates of disorganized attachment substantially declined for infants randomly assigned to high-quality foster care – but, by contrast, rates of DSED did not differ between infants who remained institutionalized and those in foster care. Zeanah et al. (2016, p. 992) have recently questioned whether DSED should be considered an “attachment” disorder at all, as it “may occur in the absence of attachment, in an aberrant attachment or in a healthy attachment to a subsequent foster or adoptive parent” (though see also Lyons-Ruth & Jacobvitz, 2016).

Though reliable epidemiologic data are lacking, correctly diagnosed attachment-related disorders are in all likelihood very rare in the general population. Thus, to understand attachment among the overwhelming majority of children in most populations, clinicians and practitioners should not reach for diagnostic attachment entities designed for use with children from contexts of extreme neglect or institutionalization. Neither should the disorganized attachment classification be “pressed into service” to fill a perceived gap in attachment-related diagnoses for community populations, especially prior to any attempts to validate it for such use.

Some markers of disorganization may, however, be found to enrich clinical assessment if embedded as part of a larger, more comprehensive assessment battery. For example, the continuous scale for the intensity of disorganized behavior (Main & Solomon, 1990) might prove useful. Though we do not know at present, extreme indications of disorganization might point to psychopathology with more or less severe symptoms and consequences (van IJzendoorn & Bakermans-Kranenburg, 2003). We return to this possibility in the next section.

### Attachment in clinical assessment and formulation

Though we offer cautions about the scope and interpretation of attachment assessments, the rich theoretical and conceptual work of Bowlby and Ainsworth has provided us with a solid base that can help guide clinical work. Attachment theory and research have a major role to play in supportive welfare and clinical work with children and families. And, it is targeted supportive work, much more than assessment, that actually makes a difference to child outcomes (Fuller & Nieto, 2014; Palusci & Ondersma, 2012). However, within the context of a wider approach with a family, attachment-related assessments may offer valuable information. The Strange Situation may be used to provide important confirmatory or disconfirmatory information, give extra insight into the child’s expectations about their caregiver, or nuance other knowledge about the dyad. To give another example where an attachment-related assessment has been used to good effect, Cyr and her team, as well as van IJzendoorn and colleagues, have independently proposed to use a brief, video-feedback-based parenting intervention that lowers rates of disorganized behaviors (see next section) as itself a tool to examine whether a parent is able to profit from further parenting support or not. A decision to treat the clinical problems within the family or place the child in out-of-home care (in a stable alternative family) may be informed by the outcome of this dynamic assessment procedure, leading to a closer connection between assessment and treatment (see Bakermans-Kranenburg, Juffer, & van IJzendoorn, in press).

If such a dynamic assessment approach were to be found sufficiently sensitive and specific in identifying maltreatment and/or predicting psychopathology and were empirically shown to do so better than “assessment as usual,” then it could be used as a valuable instrument to inform relevant interventions. Lyons-Ruth and Jacobvitz (2016) and the National Institute for Health and Care Excellence (2016) have recently argued that work toward such a procedure is a “priority” for the field. An important spur for such work are findings suggesting diversity among children placed within the disorganized classification in terms of their antecedents and implications for development, and therefore their potential risk (see e.g. Lyons-Ruth et al., 2013; Waters & Valenzuela 1999). In a recent landmark study, Padrón et al. (2014) found evidence that some forms of disorganization (indices I–V) may be more associated with genetic or individual factors, and others (indices VI–VII) might be more associated with an infant’s adverse experiences with the caregiver. Such findings underline why the disorganized classification as a whole, developed as a research tool, is not an appropriate substitute for a validated, psychometrically sound instrument for assessing caregiver or infant behaviors serving as markers of developmental risk.

However, beyond assessments per se, attachment theory and research may greatly benefit clinicians in supporting clinical insights and clinical case formulation. Although formal attachment assessments may be too costly for regular use by practitioners, the principles derived from attachment theory can inform clinical practice (see Slade, 2004; Steele & Steele, 2008; Woolgar & Baldock, 2015; Zeanah et al., 2016). For example, it may be useful to consider whether the child can use the caregiver as a safe haven when distressed and as a secure base for exploration (e.g. Powell et al., 2013). If not, supportive work can be targeted accordingly. That disorganized attachment is relationship specific also implies that clinicians need to observe the child with all his or her caregivers in order to make a more informed set of recommendations in the best interests of the child, consistent with good child welfare practice.

The idea of a paradoxical situation could also inform the practitioner’s thinking about a child’s predicament. For instance, it may be crucial for thinking about why a child who has been maltreated may still cling to his or her caregiver and be distressed by a separation, behavior that might otherwise lead a practitioner to think that the relationship between child and caregiver is robust and healthy. Practitioners can additionally learn a lot from looking at research on the intergenerational transmission of attachment (e.g. van IJzendoorn, 1995; Verhage et al., 2016). Caregivers who represent their own attachment history in a particular way on the Adult Attachment Interview (Main, Goldwyn, & Hesse, 2003), say, have flexible attention and produce a coherent narrative, tend to have children who come to display attachment behaviors to the caregiver in similar ways (i.e. can use him/her as a secure base from which to explore and a safe haven when alarmed). This link across generations appears to be at least partly explained by aspects of caregiving behavior and the caregiver’s representation of his/her child (Madigan, Hawkins, Plamondon, Moran, & Benoit, 2015).

The intergenerational “cycle of abuse” is a similar case in point, referring to a connection between a parent’s own history of abuse and an increased probability that the parent will maltreat his or her own child. This connection has been firmly established in empirical research at the group level (see meta-analysis by Schofield, Lee, & Merrick, 2013). Yet, research is clear that not every parent who was abused as a child will abuse his or her own child. Rather, studies have reported that about 30% of those who had been maltreated go on to abuse or neglect their children, which is twice the proportion of maltreatment present in families without a history of abuse matched for age, income, class, and race (Ben-David, Jonson-Reid, Drake, & Kohl, 2015; Widom, Czaja, & DuMont, 2015). Attachment theory can offer important insights into the cycle of abuse. For instance, Thompson’s (2006) research with mothers who had experienced sexual or physical violence as children found that they were substantially more likely to have a child known to social services. However, the link between the generations was not direct, as would follow from the idea that the abuse occurs through direct imitation. Instead, experiencing abuse as a child predicted less supportive relationships as an adult and greater likelihood of experiencing physical violence as an adult. These two factors in turn predicted the likelihood that their child would experience abuse from the mother or her partner.

We have also learned how the cycle of abuse can be broken, and this highlights factors that are important for practitioners. In the Minnesota study of child development and adaptation (Egeland, Jacobvitz, & Sroufe, 1988), there were three factors that jointly accounted for interruptions in the cycle of abuse. One was that many of these parents received help as children from non-abusive adults; the second was that they received therapy at some point in their lives; and the third was that the abused person entered into a supportive and non-abusive spousal relationship in adulthood (see also Thornberry et al., 2013). Those three relational themes taken together were a powerful reparative trio (Egeland, 1991). None of the caregivers who had received support from a non-abusive adult during their childhood or who had received at least a year of therapy went on to maltreat their child in this sample. Dixon, Browne, and Hamilton-Giachritsis (2009) likewise found that social supports markedly reduce the likelihood of the intergenerational cycle of abuse but, in addition, found that financial stability played an important protective role in supporting parent–child relationships. Similarly, it is notable that Loman and Siegel (2012) document substantial effects on family safety even a decade after an anti-poverty service, provided to families as part of involvement with social services, which assured that the family had reliable funds for food, clothing, and housing. However, meta-analytic data suggest that in order to be reliably successful in preventing child maltreatment, interventions may have to include parent training, and not just support (Euser, Alink, Stoltenborgh, Bakermans-Kranenburg, & van IJzendoorn, 2015).

Therefore, what we know about breaking the cycle of abuse is that even if parents have a maltreatment history, they can be helped to establish healthier relationships with their own children. Important factors include having a relationship with a therapist or other professional (e.g. as part of a parent training program), providing services to help them with financial stability, facilitating supportive relationships with a partner or friends, and helping them to identify patterns of behavior that may alarm their child or that reduce their capacity to meet their child’s needs. Thus, even though research shows an intergenerational cycle of abuse at a probabilistic group level, it is very far from destiny at the individual level. We know that families can be helped, and attachment theory is a powerful framework for guiding that help.

### Attachment-based clinical interventions

Attachment theory has been an important, guiding framework for designing specific clinical and child welfare interventions (see Steele and Steele, in press). We give four examples below to show the range of interventions, and also the diverse learning that has come from them.

First, Child-Parent Psychotherapy (CPP) is a weekly 10–12 month attachment theory-informed intervention that utilizes joint sessions between the caregiver and their young child to promote protective caregiving and secure attachment, and to target maladaptive attributions between parent and child (Lieberman, Gosh Ippen, & Van Horn, 2015). CPP has been provided to families from diverse socioeconomic and cultural backgrounds and to families confronted with child maltreatment, domestic violence, and parental depression. CPP strives to restore the caregiver into the protective shield role and to help both parent and child better regulate their emotions related to traumatic experiences, creating a shared narrative regarding the child’s experiences. Numerous positive child outcomes have been obtained in randomized controlled trials, and CPP was the first intervention to demonstrate that disorganized attachment was modifiable (Cicchetti, Rogosch, & Toth, 2006; Toth, Rogosch, Manly, & Cicchetti, 2006).

Second, Dozier and colleagues (Bernard et al., 2012) have developed a 10-session, manualized at-home intervention, the Attachment and Biobehavioral Catch-up (or ABC) program, for caregivers who are at very high risk for abusing or neglecting their children. This intervention targets three domains of caregiving, helping caregivers to (1) be nurturing when their child is distressed (e.g. to pick up a crying baby), (2) follow the child’s lead (which in turn aids children in developing regulatory capabilities), and (3) avoid displaying frightening behaviors. A distinctive feature of this intervention is the active and central role taken by caregiving coaches, who provide frequent and specific comments “in the moment.” In randomized controlled trials, the ABC intervention has been shown to facilitate parental sensitivity and child regulatory capabilities, as well as to substantially reduce rates of disorganized attachment (Bernard et al., 2012). These intervention effects have now been replicated at multiple sites (Dozier & Bernard, 2017).

Third, the Video-feedback Intervention to promote Positive Parenting and Sensitive Discipline (VIPP-SD: Juffer, Bakermans-Kranenburg, & van IJzendoorn, 2005, 2017) is also an evidence-based, short-term intervention (usually six sessions). VIPP-SD is based on an integration of attachment theory and social learning theory, particularly coercion theory (Patterson, 1982). The program is both standardized and individualized, meaning that interveners work from a standard protocol but attune the guidelines from the protocol to the parent–child dyad, resulting in individualized video feedback. Video enables precise observation of even subtle child and parent behaviors, and by providing “subtitles” to the child’s emotions and behavior shown on the film, parents are stimulated to take the child’s perspective. As a result, their observational skills improve, which is an essential element of parental sensitivity. Moreover, positive moments of parent–child interaction are reinforced by stilling and repeating such important episodes. The VIPP has been found effective (in randomized controlled trials) in improving parental sensitivity and in lowering rates of disorganized – and its later equivalent “controlling” – child attachment, especially in at-risk populations (Juffer et al., 2005, 2017; Moss et al., 2011). Training workshops are being offered on a regular basis in several countries.

Fourth, with pilot results in print (Steele, Murphy, & Steele, 2010), and a randomized controlled trial recently completed (Murphy et al., 2015), the Group Attachment-Based Intervention (GABI) is another promising manualized intervention targeting trauma- and poverty-exposed families with children aged zero to three. GABI runs for 26 weeks, and the families meet three times each week in a group over 2 h. This intervention includes parents-and-children, parents-only, and children-only components in each 120-min session. The families enrolled are quite extreme in terms of risk. Besides parental trauma and poverty, domestic and neighborhood violence, health disparities, and inability to find an adequate place to live are parts of these families’ reality. Although intensive, GABI is cost-effective because it is delivered in a group, and it combats the social isolation and poor impulse control, endemic among these families, while working on key qualities of parent–child relationships. The recently completed randomized-control trial has reported significant improvements for GABI in maternal sensitivity, child engagement, and dyadic security in free-play observations at baseline compared to end-of-treatment. This was in contrast to no gains in the treatment as usual comparison group who received parenting education classes only (M. Steele, 2017).

These supportive interventions have all demonstrated – in randomized controlled trials – that the caregiving conditions contributing to (or maintaining) disorganized attachment can be changed even among very high-risk families (for meta-analytic results, see Facompré et al., 2017). Additionally, they have helped us understand important therapeutic mechanisms that can be used by clinicians and child welfare practitioners outside of manualized interventions. These include helping caregivers to follow the child’s lead, avoid alarming behavior and provide nurturance, make sense of traumatic experiences, break social isolation, and learn strategies to remain with the child in the moment rather than become lost in memories. The question of which components are particularly effective is a topic of great significance, and, together with the National Institute for Health and Care Excellence or NICE (2016), we encourage funding for further work in this area. For an infant, the parent is the world, so by changing the behavior of the parent, we change the infant’s world. This in turn enables a transformation of the child’s behavioral regulation and sense of confidence in the caregiver. That this can often be effectively done with short-term interventions is remarkable and should effectively counteract any misconception that child attachment – whether disorganized or not – is a fixed/static trait.

We emphasize our strong consensus on the need for supportive work for families, and we are dismayed by evidence that the thresholds for forensic assessments of families are so low, and the thresholds for receiving supportive interventions are so high. For instance in the United Kingdom, 1 in 5 children born in the 2009–10 was referred to children’s social care before their fifth birthday, and 1 in 19 received a forensic assessment for child maltreatment (Bilson & Martin, 2016). Such intense focus on investigative-forensic assessment contrasts with the slim availability of supportive services for families in the United Kingdom.

In appraising such policy and service priorities from an attachment perspective, we must highlight that child removal is itself a highly risky undertaking. Extra-parental care arrangements are often unstable over time (e.g. Berlin, Vinnerljung, & Hjern, 2011), and research shows that – likely for multiple reasons – children in unstable foster care often exhibit a developmental profile comparable to children who continue to live in maltreating environments (Lawrence, Carlson, & Egeland, 2006).

Nonetheless, adoption and stable foster home placements are effective interventions for children from families where children are at risk of serious harm, such as where there are high levels of irresolvable violence and addiction (Oosterman, Schuengel, Slot, Bullens, & Doreleijers, 2007). Thus, we concur with Bowlby (1958) that child removal and placement into stable foster or adoptive care is sometimes fully justified with the child’s best long-term interests in mind. More specifically, we believe that child removal should be undertaken if (a) there is compelling evidence of maltreatment *and* (b) a fully adequate provision of supportive services has been exhausted or can be judged with confidence to be futile. In other words, by no means should family preservation always take precedence over child removal. Attachment theory may then inform effective foster parenting as well as promote understanding of why some foster children’s behaviors may be slow to change even after a good foster relationship has been built (Dozier & Rutter, 2016).

One specific population where child removal, at the expense of parent training and sufficient social and material supports, has occurred with some frequency is among families with a parent who has been diagnosed with an intellectual disability. Research indicates that 30–50% of such families face child removal, a higher rate than for any other studied population (e.g. Booth, Booth, & McConnell, 2005). Behind these removals, there is often an assumption among practitioners that these parents with intellectual disabilities are inherently unable to provide sufficient care, that their children consequently will have attachment problems (e.g. disorganized attachment), and that there is no reason to provide interventions for the parents, because they will presumably fail to learn from them as a natural function of their learning disability (Alexius & Hollander, 2014; McConnell & Llewellyn, 2002). These assumptions run counter to well-established empirical research suggesting considerable functional differences among parents diagnosed with intellectual disabilities as well as indication that their caregiving may be responsive to supportive interventions (for reviews, see Feldman, 2010; Schuengel, Kef, Hodes, & Meppelder, 2017). In the only child attachment study conducted in this population, parental intellectual disability alone was *not* associated with either fearful/disorganized attachment representations or with other forms of insecure attachment (Granqvist, Forslund, Fransson, Springer, & Lindberg, 2014). However, the *combination* of maternal intellectual disability and the mothers having been subjected to serious forms of maltreatment during their own upbringing made it difficult for these mothers to be sensitive to their children (Lindberg et al., 2017) and predicted fearful/disorganized attachment representations (Granqvist et al., 2014).

The evidence of effectiveness of some supportive interventions for families in reducing rates of child maltreatment and disorganized attachment suggest that such interventions should be a public health priority, and an area of further investment. However, though policy that curbs maltreatment (via supportive interventions) will likely contribute toward the reduction of disorganized attachment, it will not lead to its eradication, as disorganized attachment is sometimes caused by other factors than maltreatment.

## Summary and conclusions

Disorganized behaviors can occur for a variety of reasons, and many of them do not in themselves indicate maltreatment, developmental risk, or mental illness. Under existing protocols, only when they are of sufficient intensity and occur in the caregiver’s presence in a standardized procedure (i.e. the Strange Situation) can a classification of disorganized attachment be assigned in a valid way. This is also predicated on the classification being completed by a certified coder.

Disorganized attachment is more common among children who have been maltreated. However, a substantial proportion of maltreated children do not show disorganized attachment in the Strange Situation, and many children showing disorganized attachment in the Strange Situation have not been maltreated. There are other pathways to disorganized attachment besides maltreatment. These other pathways often feature frightening, frightened, and dissociative parental behaviors, which are more common among caregivers struggling with unresolved loss/trauma or multiple compounded socioeconomic risks. Other causal conditions include major (extended or repeated) separations under adverse conditions, and congenital factors, possibly in combination with caregiver factors (Lakatos et al., 2000; Padrón et al., 2014; Spangler et al. 1996).

Empirical research at the group level has established disorganized attachment as a predictor, of small-to-moderate magnitude, for the development of social and behavior problems. However, this research is equally clear that disorganized attachment does not inevitably cause later problems. Nor is disorganized attachment a validated individual-level clinical diagnosis, unlike the two attachment disorders included in the DSM/ICD diagnostic systems – conditions originally developed for young children brought up under deprivation in institutional settings.

Misapplications of attachment theory in general, and disorganized attachment in particular, have accrued in recent years, as reflected for example in some child removal decisions. These misapplications can result from erroneous assumptions that (1) attachment measures can be used as definitive assessments of the individual in forensic/child-protection settings and that (2) disorganized attachment reliably indicates child maltreatment (3) is a strong predictor of pathology and (4) cannot be changed through interventions in the child’s original home. Such misapplications may selectively harm already underprivileged families, such as those facing multiple socioeconomic risk factors or including a parent with functional impairments. These misapplications not only violate children’s and parents’ human rights but in many cases, they may also represent discriminatory practice against minorities in need of our social and material support.

However, it is also important to recognize that attachment theory, assessments, and research can have major roles to play in clinical formulation and supportive welfare and clinical work. There is robust evidence that attachment-based interventions as well as naturalistically occurring reparative relationship experiences (stable, safe, and nurturing relationships) can break intergenerational cycles of abuse and lower the proportion of children displaying disorganized attachment. We conclude that the real practical utility of attachment theory and research resides in supporting understanding of families and in providing supportive, evidence-based interventions.
